# Poly[bis­[μ_2_-4,4′-bis­(imidazol-1-ylmeth­yl)biphenyl-κ^2^ *N*:*N*′]di­chlorido­nickel(II)]

**DOI:** 10.1107/S2414314622003777

**Published:** 2022-04-12

**Authors:** Min Deng, Yue Yin, Shan-Shan Wang, Xin-Yi Qi, Ai-Xin Zhu

**Affiliations:** a Faculty of Chemistry and Chemical Engineering, Yunnan Normal University, Kunming 650050, People’s Republic of China; Purdue University, USA

**Keywords:** crystal structure, coordination polymer, imidazole, nickel

## Abstract

In the title compound, [NiCl_2_(C_20_H_18_N_4_)_2_]_*n*_, the Ni^2+^ cation is situated on an inversion center and is coordinated by two chloride ions and four imidazole N atoms of four different 4,4′-bis­[(1*H*-imidazol-1-yl)meth­yl]-1,1′-biphenyl (BIMB), forming a slightly distorted octa­hedral geometry. Each BIMB ligand adopts a linear linker to connect Ni^2+^ ions, forming a two-dimensional layer with an **sql** network. In the crystal, neighboring layers repeat in an *ABAB* stacking mode, and weak inter­molecular C—H⋯Cl hydrogen bonds between alternate layers lead to a three-dimensional, twofold inter­penetrated, supra­molecular framework with a **pcu** topology net.

## Abstract 2

The crystal structure of a two-dimensional metal–organic compound constructed from 4,4′-bis­[(1*H*-imidazol-1-yl)meth­yl]-1,1′-biphenyl (BIMB) and nickel ions is described. Each BIMB ligand adopts a linear linker to connect Ni^2+^ ions, forming a layer with an **sql** network. In the crystal, neighboring layers repeat in an *ABAB* stacking mode, and weak inter­molecular C—H⋯Cl hydrogen bonds between alternate layers lead to a three-dimensional, twofold inter­penetrated, supra­molecular framework with a **pcu** topology net.

## Graphical abstract

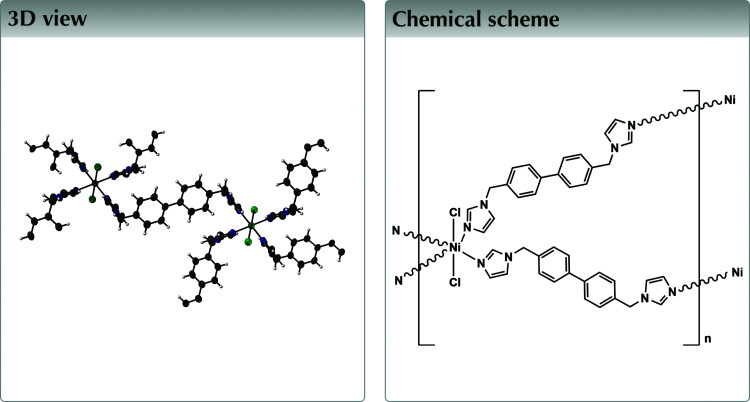

## Structure description

Over the last two decades, imidazole and its derivatives have attracted a lot of attention as *N*-heterocyclic aromatic ligands, since they can easily form metal–imidazole frameworks with special luminescent, magnetic and favorable gas-adsorption abilities (Banerjee *et al.* 2008 ▸; Zhang *et al.* 2012 ▸; Zhu *et al.* 2012 ▸; Chen *et al.* 2014 ▸). As an extended imidazole-type linker, the flexible ligand 4,4′-bis­[(1*H*-imidazol-1-yl)meth­yl]-1,1′-biphenyl (BIMB) exhibits a geometrical diversity with *cis* or *trans* conformations, leading to diverse structures of coordination compounds. Until now, most reported metal–organic compounds based on BIMB ligands have employed organic multi­carboxyl­ates as co-ligands because BIMB is a neutral ligand and another anion is needed to balance the charge requirement to form a neutral framework. Common inorganic anions such as Cl^−^, Br^−^, I^−^, NO_3_^−^, SO_4_^2−^, N_3_^−^, *etc*. can also be used as co-ligands to balance the charge requirement. However, only ten examples of neutral, BIMB-based metal–organic compounds have been reported [according to the Cambridge Structural Database (CSD, Version 5.43 with update of March, 2022; Groom *et al.*, 2016 ▸) with inorganic anions as co-ligands.

**Figure 1:**
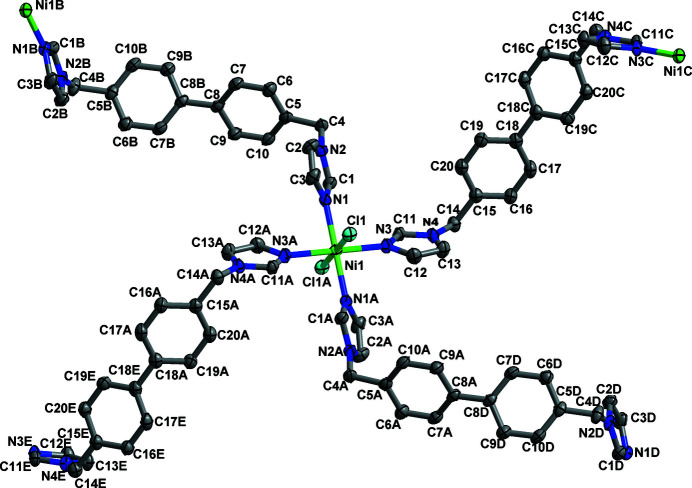
The coordination environment of the zinc ions and the BIMB ligands in the title compound, with displacement ellipsoids drawn at the 30% probability level. H atoms are omitted for clarity. [Symmetry codes: (A) 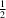 − *x*, 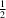 − *y*, 1 − *z*; (B) −*x*, *y*, 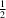 − *z*; (C) 1 − *x*, *y*, 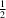 − *z*; (D) 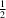 + *x*, 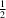 − *y*, 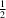 + *z*; (E) *x* − 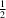 , 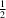 − *y*, 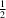 + *z*.]

**Figure 2:**
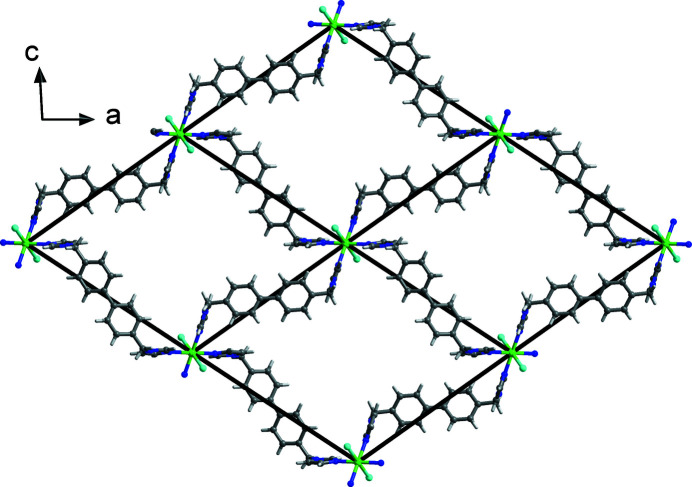
The two-dimensional structure of the title compound with **sql** network viewed along the *b* axis.

**Figure 3:**
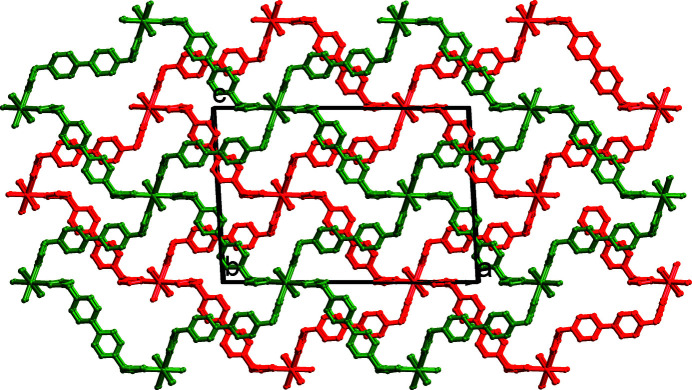
The packing of the title compound viewed along the *b* axis. H atoms are omitted for clarity.

**Figure 5:**
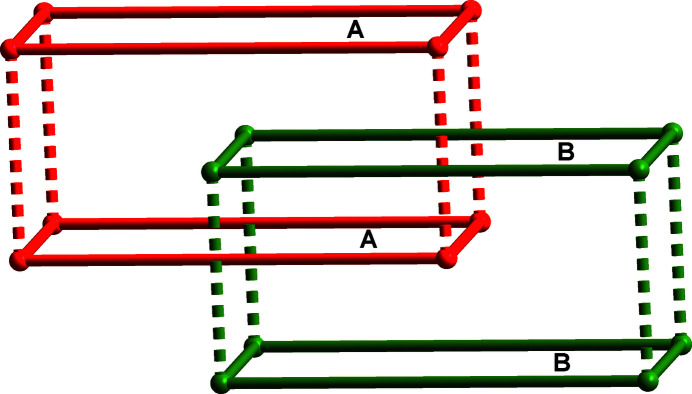
The twofold inter­penetrated supra­molecular framework with a **pcu** topology network connected by C—H⋯Cl hydrogen bonds (shown as dashed lines).

The asymmetric unit of the title compound, [NiCl_2_(C_20_H_18_N_4_)_2_]_*n*_, contains one half nickel(II) ion, two half BIMB ligands and one chloride ion (Fig. 1 ▸). The nickel(II) ion sits on an inversion center and is coordinated by four imidazole nitro­gen atoms from four different BIMB ligands [Ni—N = 2.100 (3)–2.108 (3) Å] and two chloride ions [Ni—Cl = 2.4793 (11) Å], forming a slightly distorted octa­hedral geometry. In the crystal, the BIMB ligands have twofold rotational symmetry, being bis­ected by rotation axes, and the biphenyl groups are not coplanar, with dihedral angles of 33.21 (10) and 35.4 (10)° between the ring planes. The dihedral angles between the imidazole ring plane and the average plane of the biphenyl group are 87.71 (14) and 81.93 (14)°. Each BIMB ligand exhibits a *cis*-conformation relative to the average plane of the biphenyl group, and acts as a linear linker between Ni^2+^ ions, which gives a corrugated two-dimensional layer structure with an **sql** (square lattice) network as illustrated in Fig. 2 ▸. The layers stack in an –*ABAB*– mode, and the Ni^2+^ ion in one layer is located at the center of the grid of adjacent layers. Thus, there are no residual solvent-accessible voids in this compound. Alternate layers between *A*—*A* or *B*—*B* layers are further linked by C—H⋯Cl hydrogen bonds (Table 1 ▸, Figs. 3 ▸ and 4 ▸) to form a three-dimensional, twofold inter­penetrated, supra­molecular framework with a **pcu** (primitive cubic) topology network (Fig. 5 ▸).

The structure of the title compound is isomorphous to that of the cadmium(II) compound, whose structure has been studied at 200 K (Zhao *et al.* 2003 ▸). This structural similarity of the Cd^II^ and Ni^II^ compounds is somewhat unexpected in view of the different effective radii of these ions (Shannon & Prewitt, 1969 ▸, 1970 ▸), which causes the differences between *M*—N distances [Cd—-N = 2.339 (2)–2.364 (2) Å in the cadmium(II) compound]. It should also be noted that the title compound was easily obtained within one day using solvothermal conditions, whereas the cadmium(II) compound was obtained after several weeks using a slow-diffusion method.

## Synthesis and crystallization

A mixture of NiCl_2_·H_2_O (24 mg, 0.1 mmol), BIMB (62 mg, 0.2 mmol) and DMF (6 ml) was added to a 20 ml glass vial and then ultrasonicated for 1 minute. The vial was capped tightly and placed in an oven at 120°C. After 12 h, the vial was removed from the oven and allowed to cool to room temperature. The light-green transparent needle-like crystals were collected by filtration, washed with DMF and dried under ambient conditions. About 34 mg of product was obtained (44% yield based on BIMB ligand). The phase purity of the bulk sample was verified by powder X-ray diffraction (PXRD). The experimental and simulated powder XRD patterns of the title compound are displayed in Fig. S1 of the supporting information. Their peak positions are in good agreement with each other, indicating the phase purity of the title compound (slight intensity mismatches due to preferred orientation are observed).

## Refinement

Crystal data, data collection and structure refinement details are summarized in Table 2 ▸.

## Supplementary Material

Crystal structure: contains datablock(s) I. DOI: 10.1107/S2414314622003777/zl4050sup1.cif

Structure factors: contains datablock(s) I. DOI: 10.1107/S2414314622003777/zl4050Isup3.hkl

The experimental and simulated powder XRD patterns of the title compound. DOI: 10.1107/S2414314622003777/zl4050sup4.docx

CCDC reference: 2164744

Additional supporting information:  crystallographic information; 3D view; checkCIF report

## Figures and Tables

**Figure 4:**
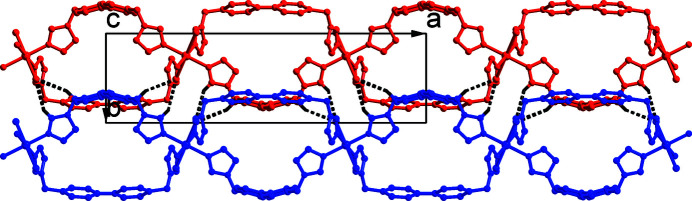
View of the C—H⋯Cl hydrogen bonds (dashed lines) between alternate layers along the *c* axis. H atoms not involved in hydrogen bonding are omitted.

**Table 1:** Hydrogen-bond geometry (Å, °)

*D*—H⋯*A*	*D*—H	H⋯*A*	*D*⋯*A*	*D*—H⋯*A*
C11—H11⋯Cl1^i^	0.93	2.79	3.605 (4)	147
C14—H14*B*⋯Cl1^i^	0.97	2.80	3.686 (5)	153

Symmetry code: (i) 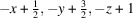 .

**Table 2:** Experimental details

Crystal data	
Chemical formula	[NiCl_2_(C_20_H_18_N_4_)_2_]
*M*_r_	758.38
Crystal system, space group	Monoclinic, *C*2/*c*
Temperature (K)	296
*a*, *b*, *c* (Å)	26.453 (3), 7.3571 (7), 18.099 (2)
β (°)	93.223 (11)
*V* (Å^3^)	3516.8 (7)
*Z*	4
Radiation type	Mo *K*α
μ (mm^−1^)	0.75
Crystal size (mm)	0.30 × 0.22 × 0.16

Data collection	
Diffractometer	Oxford Diffraction, Xcalibur, Eos, Gemini
Absorption correction	Multi-scan (*CrysAlis PRO*; Rigaku OD, 2015 ▸)
*T*_min_, *T*_max_	0.856, 1.000
No. of measured, independent and observed [*I* > 2σ(*I*)] reflections	16025, 4343, 2543
*R*_int_	0.078
(sin θ/λ)_max_ (Å^−1^)	0.692

Refinement	
*R*[*F* ^2^ > 2σ(*F* ^2^)], *wR*(*F* ^2^), *S*	0.072, 0.161, 1.06
No. of reflections	4343
No. of parameters	232
H-atom treatment	H-atom parameters constrained
Δρ_max_, Δρ_min_ (e Å^−3^)	0.61, −0.27

Computer programs: *CrysAlis PRO* (Rigaku OD, 2015 ▸), *SHELXT2014/5* (Sheldrick, 2015*a* ▸), *SHELXL2018/3* (Sheldrick, 2015*b* ▸) and *DIAMOND* (Brandenburg, 1999 ▸).

## full crystallographic data

### Crystal data

**Table:**

[NiCl_2_(C_20_H_18_N_4_)_2_]	*F*(000) = 1576
*M**_r_* = 758.38	*D*_x_ = 1.432 Mg m^−^^3^
Monoclinic, *C*2/*c*	Mo *K*α radiation, λ = 0.71073 Å
*a* = 26.453 (3) Å	Cell parameters from 3041 reflections
*b* = 7.3571 (7) Å	θ = 2.7–22.6°
*c* = 18.099 (2) Å	µ = 0.75 mm^−^^1^
β = 93.223 (11)°	*T* = 296 K
*V* = 3516.8 (7) Å^3^	Needle, green
*Z* = 4	0.30 × 0.22 × 0.16 mm

### Data collection

**Table:**

Oxford Diffraction, Xcalibur, Eos, Gemini diffractometer	2543 reflections with *I* > 2σ(*I*)
Radiation source: fine-focus sealed X-ray tube	*R*_int_ = 0.078
ω scans	θ_max_ = 29.5°, θ_min_ = 2.3°
Absorption correction: multi-scan (CrysAlisPro; Rigaku OD, 2015)	*h* = −35→35
*T*_min_ = 0.856, *T*_max_ = 1.000	*k* = −9→10
16025 measured reflections	*l* = −24→19
4343 independent reflections	

### Refinement

**Table:**

Refinement on *F*^2^	Primary atom site location: structure-invariant direct methods
Least-squares matrix: full	Secondary atom site location: difference Fourier map
*R*[*F*^2^ > 2σ(*F*^2^)] = 0.072	Hydrogen site location: inferred from neighbouring sites
*wR*(*F*^2^) = 0.161	H-atom parameters constrained
*S* = 1.06	*w* = 1/[σ^2^(*F*_o_^2^) + (0.0438*P*)^2^ + 5.351*P*] where *P* = (*F*_o_^2^ + 2*F*_c_^2^)/3
4343 reflections	(Δ/σ)_max_ < 0.001
232 parameters	Δρ_max_ = 0.61 e Å^−^^3^
0 restraints	Δρ_min_ = −0.27 e Å^−^^3^

### Special details

**Table:**

Geometry. All esds (except the esd in the dihedral angle between two l.s. planes) are estimated using the full covariance matrix. The cell esds are taken into account individually in the estimation of esds in distances, angles and torsion angles; correlations between esds in cell parameters are only used when they are defined by crystal symmetry. An approximate (isotropic) treatment of cell esds is used for estimating esds involving l.s. planes.
Refinement. All H atoms were placed in idealized positions (C—H = 0.93 Å for aromatic H; C—H = 0.97 Å for methylene H) and refined as riding atoms with *U*_iso_(H) = 1.2*U*_eq_(C).

### Fractional atomic coordinates and isotropic or equivalent isotropic displacement parameters (Å^2^)

**Table:**

	*x*	*y*	*z*	*U*_iso_*/*U*_eq_	
Ni1	0.250000	0.250000	0.500000	0.0419 (2)	
Cl1	0.21866 (4)	0.51154 (14)	0.57071 (6)	0.0488 (3)	
N1	0.22244 (12)	0.3739 (5)	0.40075 (19)	0.0423 (8)	
N2	0.20188 (12)	0.5693 (5)	0.31205 (19)	0.0454 (9)	
N3	0.32078 (12)	0.3777 (5)	0.49579 (19)	0.0451 (9)	
N4	0.38019 (12)	0.5864 (5)	0.4895 (2)	0.0454 (9)	
C1	0.21920 (15)	0.5462 (6)	0.3818 (2)	0.0480 (11)	
H1	0.228055	0.641502	0.413725	0.058*	
C2	0.19229 (17)	0.4035 (7)	0.2839 (3)	0.0605 (13)	
H2	0.179357	0.375818	0.236414	0.073*	
C3	0.20524 (17)	0.2848 (7)	0.3387 (3)	0.0594 (13)	
H3	0.202711	0.159098	0.334459	0.071*	
C4	0.19061 (16)	0.7409 (7)	0.2746 (3)	0.0598 (13)	
H4A	0.205060	0.740434	0.226555	0.072*	
H4B	0.205927	0.839897	0.303368	0.072*	
C5	0.13415 (16)	0.7708 (6)	0.2650 (3)	0.0485 (11)	
C6	0.10774 (17)	0.7322 (6)	0.1991 (3)	0.0555 (12)	
H6	0.125207	0.697958	0.158149	0.067*	
C7	0.05550 (17)	0.7438 (6)	0.1933 (3)	0.0511 (11)	
H7	0.038471	0.716996	0.148230	0.061*	
C8	0.02803 (15)	0.7940 (5)	0.2523 (2)	0.0443 (11)	
C9	0.05500 (17)	0.8394 (7)	0.3182 (3)	0.0568 (12)	
H9	0.037767	0.877617	0.358795	0.068*	
C10	0.10719 (17)	0.8279 (7)	0.3234 (3)	0.0601 (13)	
H10	0.124556	0.859617	0.367591	0.072*	
C11	0.33046 (15)	0.5513 (6)	0.4876 (2)	0.0460 (11)	
H11	0.305554	0.639986	0.481224	0.055*	
C12	0.36761 (17)	0.2989 (6)	0.5055 (3)	0.0573 (13)	
H12	0.373160	0.175661	0.513987	0.069*	
C13	0.40491 (18)	0.4252 (6)	0.5009 (3)	0.0580 (13)	
H13	0.439701	0.405819	0.504830	0.070*	
C14	0.40289 (17)	0.7651 (6)	0.4815 (3)	0.0523 (12)	
H14A	0.425099	0.790809	0.524742	0.063*	
H14B	0.376426	0.856560	0.478823	0.063*	
C15	0.43247 (16)	0.7761 (6)	0.4137 (3)	0.0474 (11)	
C16	0.48277 (16)	0.8245 (6)	0.4154 (3)	0.0558 (12)	
H16	0.499348	0.852339	0.460692	0.067*	
C17	0.50950 (17)	0.8329 (7)	0.3518 (3)	0.0588 (13)	
H17	0.543335	0.867828	0.354691	0.071*	
C18	0.48594 (16)	0.7894 (5)	0.2841 (2)	0.0468 (11)	
C19	0.43553 (17)	0.7414 (6)	0.2819 (3)	0.0544 (12)	
H19	0.418879	0.713521	0.236701	0.065*	
C20	0.40927 (17)	0.7340 (6)	0.3457 (3)	0.0556 (12)	
H20	0.375352	0.700011	0.342800	0.067*	

### Atomic displacement parameters (Å^2^)

**Table:**

	*U*^11^	*U*^22^	*U*^33^	*U*^12^	*U*^13^	*U*^23^
Ni1	0.0420 (4)	0.0360 (4)	0.0478 (5)	0.0039 (3)	0.0020 (3)	0.0062 (4)
Cl1	0.0494 (6)	0.0406 (6)	0.0563 (7)	0.0077 (5)	0.0024 (5)	0.0018 (5)
N1	0.0364 (19)	0.042 (2)	0.048 (2)	−0.0009 (16)	−0.0007 (16)	0.0031 (17)
N2	0.0367 (19)	0.050 (2)	0.049 (2)	0.0005 (17)	−0.0055 (16)	0.0120 (18)
N3	0.0380 (19)	0.039 (2)	0.059 (2)	0.0035 (16)	0.0041 (17)	0.0061 (18)
N4	0.040 (2)	0.036 (2)	0.060 (2)	−0.0003 (16)	0.0051 (17)	0.0076 (17)
C1	0.046 (3)	0.047 (3)	0.050 (3)	−0.002 (2)	−0.007 (2)	0.000 (2)
C2	0.060 (3)	0.060 (3)	0.059 (3)	0.014 (3)	−0.016 (2)	−0.005 (3)
C3	0.055 (3)	0.046 (3)	0.075 (4)	0.009 (2)	−0.013 (3)	−0.005 (3)
C4	0.044 (3)	0.065 (3)	0.069 (3)	−0.003 (2)	−0.004 (2)	0.027 (3)
C5	0.045 (2)	0.045 (3)	0.055 (3)	0.000 (2)	−0.005 (2)	0.015 (2)
C6	0.052 (3)	0.056 (3)	0.058 (3)	0.005 (2)	−0.004 (2)	0.002 (2)
C7	0.053 (3)	0.051 (3)	0.048 (3)	−0.001 (2)	−0.013 (2)	−0.002 (2)
C8	0.048 (2)	0.037 (2)	0.046 (3)	−0.0001 (18)	−0.009 (2)	0.004 (2)
C9	0.053 (3)	0.068 (3)	0.048 (3)	0.002 (2)	−0.006 (2)	−0.001 (3)
C10	0.052 (3)	0.072 (3)	0.055 (3)	−0.002 (3)	−0.018 (2)	0.001 (3)
C11	0.037 (2)	0.045 (3)	0.056 (3)	0.0067 (19)	0.001 (2)	0.006 (2)
C12	0.055 (3)	0.040 (3)	0.077 (4)	0.008 (2)	0.007 (3)	0.012 (2)
C13	0.049 (3)	0.046 (3)	0.079 (4)	0.008 (2)	0.002 (2)	0.004 (3)
C14	0.051 (3)	0.046 (3)	0.060 (3)	−0.003 (2)	0.005 (2)	−0.001 (2)
C15	0.045 (2)	0.040 (3)	0.057 (3)	0.001 (2)	0.003 (2)	0.001 (2)
C16	0.044 (3)	0.062 (3)	0.061 (3)	−0.004 (2)	0.000 (2)	0.000 (3)
C17	0.039 (2)	0.060 (3)	0.078 (4)	−0.006 (2)	0.005 (2)	−0.002 (3)
C18	0.046 (3)	0.032 (2)	0.062 (3)	0.0030 (18)	0.005 (2)	0.000 (2)
C19	0.048 (3)	0.058 (3)	0.057 (3)	−0.002 (2)	−0.001 (2)	−0.002 (2)
C20	0.043 (3)	0.058 (3)	0.066 (3)	−0.004 (2)	0.003 (2)	−0.001 (3)

### Geometric parameters (Å, º)

**Table:**

Ni1—N3^i^	2.100 (3)	C6—H6	0.9300
Ni1—N3	2.100 (3)	C7—C8	1.377 (6)
Ni1—N1	2.108 (3)	C7—H7	0.9300
Ni1—N1^i^	2.108 (3)	C8—C9	1.395 (6)
Ni1—Cl1	2.4793 (11)	C8—C8^ii^	1.480 (8)
Ni1—Cl1^i^	2.4793 (11)	C9—C10	1.381 (6)
N1—C1	1.315 (5)	C9—H9	0.9300
N1—C3	1.357 (5)	C10—H10	0.9300
N2—C1	1.330 (5)	C11—H11	0.9300
N2—C2	1.341 (6)	C12—C13	1.361 (6)
N2—C4	1.456 (5)	C12—H12	0.9300
N3—C11	1.312 (5)	C13—H13	0.9300
N3—C12	1.370 (5)	C14—C15	1.495 (6)
N4—C11	1.339 (5)	C14—H14A	0.9700
N4—C13	1.365 (5)	C14—H14B	0.9700
N4—C14	1.456 (5)	C15—C16	1.376 (6)
C1—H1	0.9300	C15—C20	1.378 (6)
C2—C3	1.351 (6)	C16—C17	1.386 (6)
C2—H2	0.9300	C16—H16	0.9300
C3—H3	0.9300	C17—C18	1.380 (6)
C4—C5	1.510 (6)	C17—H17	0.9300
C4—H4A	0.9700	C18—C19	1.378 (6)
C4—H4B	0.9700	C18—C18^iii^	1.476 (9)
C5—C10	1.374 (6)	C19—C20	1.382 (6)
C5—C6	1.377 (6)	C19—H19	0.9300
C6—C7	1.383 (6)	C20—H20	0.9300
			
N3^i^—Ni1—N3	180.0	C7—C6—H6	119.7
N3^i^—Ni1—N1	87.59 (13)	C8—C7—C6	121.8 (4)
N3—Ni1—N1	92.41 (13)	C8—C7—H7	119.1
N3^i^—Ni1—N1^i^	92.41 (13)	C6—C7—H7	119.1
N3—Ni1—N1^i^	87.59 (13)	C7—C8—C9	117.4 (4)
N1—Ni1—N1^i^	180.00 (17)	C7—C8—C8^ii^	121.8 (4)
N3^i^—Ni1—Cl1	90.22 (10)	C9—C8—C8^ii^	120.8 (5)
N3—Ni1—Cl1	89.78 (10)	C10—C9—C8	120.3 (5)
N1—Ni1—Cl1	89.69 (10)	C10—C9—H9	119.8
N1^i^—Ni1—Cl1	90.31 (10)	C8—C9—H9	119.8
N3^i^—Ni1—Cl1^i^	89.78 (10)	C5—C10—C9	121.8 (4)
N3—Ni1—Cl1^i^	90.22 (10)	C5—C10—H10	119.1
N1—Ni1—Cl1^i^	90.31 (10)	C9—C10—H10	119.1
N1^i^—Ni1—Cl1^i^	89.69 (10)	N3—C11—N4	112.5 (4)
Cl1—Ni1—Cl1^i^	180.0	N3—C11—H11	123.8
C1—N1—C3	103.7 (4)	N4—C11—H11	123.8
C1—N1—Ni1	130.8 (3)	C13—C12—N3	110.9 (4)
C3—N1—Ni1	125.5 (3)	C13—C12—H12	124.6
C1—N2—C2	107.0 (4)	N3—C12—H12	124.6
C1—N2—C4	127.1 (4)	C12—C13—N4	105.0 (4)
C2—N2—C4	125.7 (4)	C12—C13—H13	127.5
C11—N3—C12	104.2 (4)	N4—C13—H13	127.5
C11—N3—Ni1	128.3 (3)	N4—C14—C15	111.6 (4)
C12—N3—Ni1	127.4 (3)	N4—C14—H14A	109.3
C11—N4—C13	107.3 (4)	C15—C14—H14A	109.3
C11—N4—C14	125.6 (4)	N4—C14—H14B	109.3
C13—N4—C14	127.1 (4)	C15—C14—H14B	109.3
N1—C1—N2	112.6 (4)	H14A—C14—H14B	108.0
N1—C1—H1	123.7	C16—C15—C20	117.4 (4)
N2—C1—H1	123.7	C16—C15—C14	123.0 (4)
N2—C2—C3	106.0 (4)	C20—C15—C14	119.6 (4)
N2—C2—H2	127.0	C15—C16—C17	122.0 (4)
C3—C2—H2	127.0	C15—C16—H16	119.0
C2—C3—N1	110.8 (4)	C17—C16—H16	119.0
C2—C3—H3	124.6	C18—C17—C16	120.1 (4)
N1—C3—H3	124.6	C18—C17—H17	120.0
N2—C4—C5	110.8 (3)	C16—C17—H17	120.0
N2—C4—H4A	109.5	C19—C18—C17	118.2 (4)
C5—C4—H4A	109.5	C19—C18—C18^iii^	120.6 (5)
N2—C4—H4B	109.5	C17—C18—C18^iii^	121.2 (5)
C5—C4—H4B	109.5	C18—C19—C20	121.1 (4)
H4A—C4—H4B	108.1	C18—C19—H19	119.4
C10—C5—C6	118.0 (4)	C20—C19—H19	119.4
C10—C5—C4	120.5 (4)	C15—C20—C19	121.1 (4)
C6—C5—C4	121.4 (5)	C15—C20—H20	119.4
C5—C6—C7	120.6 (5)	C19—C20—H20	119.4
C5—C6—H6	119.7		
			
C3—N1—C1—N2	−0.9 (5)	C12—N3—C11—N4	1.7 (5)
Ni1—N1—C1—N2	176.6 (3)	Ni1—N3—C11—N4	178.2 (3)
C2—N2—C1—N1	1.2 (5)	C13—N4—C11—N3	−1.1 (5)
C4—N2—C1—N1	175.8 (4)	C14—N4—C11—N3	179.6 (4)
C1—N2—C2—C3	−0.9 (5)	C11—N3—C12—C13	−1.7 (5)
C4—N2—C2—C3	−175.6 (4)	Ni1—N3—C12—C13	−178.2 (3)
N2—C2—C3—N1	0.4 (6)	N3—C12—C13—N4	1.1 (6)
C1—N1—C3—C2	0.3 (5)	C11—N4—C13—C12	0.0 (5)
Ni1—N1—C3—C2	−177.4 (3)	C14—N4—C13—C12	179.3 (4)
C1—N2—C4—C5	−105.6 (5)	C11—N4—C14—C15	−116.3 (5)
C2—N2—C4—C5	68.0 (6)	C13—N4—C14—C15	64.6 (6)
N2—C4—C5—C10	77.7 (6)	N4—C14—C15—C16	−123.9 (5)
N2—C4—C5—C6	−98.6 (5)	N4—C14—C15—C20	55.2 (5)
C10—C5—C6—C7	−2.6 (7)	C20—C15—C16—C17	0.6 (7)
C4—C5—C6—C7	173.8 (4)	C14—C15—C16—C17	179.7 (4)
C5—C6—C7—C8	0.0 (7)	C15—C16—C17—C18	−1.0 (7)
C6—C7—C8—C9	2.2 (7)	C16—C17—C18—C19	1.2 (7)
C6—C7—C8—C8^ii^	−175.6 (3)	C16—C17—C18—C18^iii^	−177.3 (3)
C7—C8—C9—C10	−2.0 (7)	C17—C18—C19—C20	−1.0 (7)
C8^ii^—C8—C9—C10	175.9 (4)	C18^iii^—C18—C19—C20	177.5 (3)
C6—C5—C10—C9	2.8 (7)	C16—C15—C20—C19	−0.4 (7)
C4—C5—C10—C9	−173.6 (4)	C14—C15—C20—C19	−179.5 (4)
C8—C9—C10—C5	−0.5 (8)	C18—C19—C20—C15	0.7 (7)

Symmetry codes: (i) −*x*+1/2, −*y*+1/2, −*z*+1; (ii) −*x*, *y*, −*z*+1/2; (iii) −*x*+1, *y*, −*z*+1/2.

### Hydrogen-bond geometry (Å, º)

**Table:**

*D*—H···*A*	*D*—H	H···*A*	*D*···*A*	*D*—H···*A*
C11—H11···Cl1^iv^	0.93	2.79	3.605 (4)	147
C14—H14*B*···Cl1^iv^	0.97	2.80	3.686 (5)	153

Symmetry code: (iv) −*x*+1/2, −*y*+3/2, −*z*+1.
