# Supplementary material for: Poly[bis­[μ2-4,4′-bis­(imidazol-1-ylmeth­yl)biphenyl-κ2 N:N′]di­chlorido­nickel(II)]
Source: IUCrdata. 2022 Apr 12;7(Pt 4):x220377. doi: 10.1107/S2414314622003777 (PMC9462028; doi:10.1107/S2414314622003777)
Supplement: Supplementary file 3 [file x-07-x220377-sup4.docx]

The phase-purity of the title compound was checked by powder X-ray diffraction (PXRD) using a MiniFlex600 automated diffractometer (Cu-Kα, λ = 1.5418 Å).





Figure S1. The experimental and simulated powder XRD patterns of the title compound.
